# Monthly Variations in Perfluorinated Compound Concentrations in Groundwater

**DOI:** 10.3390/toxics6030056

**Published:** 2018-09-14

**Authors:** Megan Steele, Converse Griffith, Christin Duran

**Affiliations:** 1 UES, Force Health Branch, United States Air Force School of Aerospace Medicine, 711 Human Performance Wing, Dayton, OH 45431, USA; megan.steele.1.ctr@us.af.mil (M.S.); converse.griffith.ctr@us.af.mil (C.G.); 2 Force Health Branch, United States Air Force School of Aerospace Medicine, 711 Human Performance Wing, Dayton, OH 45433, USA

**Keywords:** PFAS, PFOS, PFOA, perfluorooctane sulfonate, perfluorooctanoic acid

## Abstract

Large-scale manufacturing of poly- and perfluorinated compounds in the second half of the 20th century has led to their ubiquity in the environment, and their unique structure has made them persistent contaminants. A recent drinking water advisory level issued by the United States Environmental Protection Agency lowered the advisory level concentration of perfluorooctanesulfonic acid (PFOS) and perfluorooctanoic acid (PFOA) from 200 nanograms per liter and 400 nanograms per liter, respectively, to 70 nanograms per liter separately or combined. Small temporal variations in PFOS and PFOA concentrations could be the difference between meeting or exceeding the recommended limit. In this study, newly sampled data from a contaminated military site in Alaska and historical data from former Pease Air Force Base were collected. Data were evaluated to determine if monthly variations within PFOS and PFOA existed. No statistically significant temporal trend was observed in the Alaska data, while the results from Pease, although statistically significant, showed the spread of observed contaminant concentrations around the fitted line is broad (as indicated by the low *R*^2^ values), indicating that collection date has little value in predicting contaminant concentrations. Though not currently the subject of a US EPA health advisory, data on perfluorobutanesulfonic acid (PFBS), perfluorohexane sulfonic acid (PFHxS), perfluoroheptanoic acid (PFHpA), and perfluorononanoic acid (PFNA) were collected for each site and their average concentrations evaluated.

## 1. Introduction

Per- and polyfluoroalkyl substances (PFAS) have a carbon chain backbone bonded to either fluorine or functional groups [1]. This structure makes PFAS both hydrophobic and oleophobic, properties that are essential for making stain- and stick-resistant products, such as fast food wrappers and stain-resistant fabric [2]. The wetting properties that allow these compounds to evenly coat a surface make them ideal additives for aqueous firefighting foam (AFFF), since unbroken coverage of spilled fuel can be achieved through a relatively small amount of PFAS [3]. At 105.4 kcal/mol, the carbon-fluorine bond is one of the strongest covalent bonds, making the compounds highly resistant to change by chemical or physical means [4,5]. While this bond property is advantageous in high-temperature or chemically aggressive processes, it makes PFAS persistent organic pollutants once they have reached the environment [6].

While an entire class of PFAS exist, two specific compounds have been singled out for attention: Perfluorooctanoic acid (PFOA) and perfluorooctanesulfonic acid (PFOS). These two compounds are ubiquitous, present in 95% of American citizens’ serum [7], and found at low levels in the soil of Antarctica [8]. Both of these compounds have been observed to bioaccumulate in birds [9], and mammals [10] while PFOS is shown to bioaccumlate in fish though PFOA is not [11]. Just as they are long lasting in the environment, they persist within the serum of exposed humans. A five-year study of former workers from a PFAS production facility found the half-life of PFOA and PFOS in humans to be 3.5 years and 4.8 years, respectively, with an average serum concentration of 799 ng/mL for PFOS and 691 ng/mL for PFOA [12,13]. Human exposure to PFAS has been shown to occur through air, drinking water, food, and indoor dust from treated furniture [14].

PFOS has been internationally recognized as a persistent organic pollutant under the Stockholm Convention since 2009 [15], and though no decision has been reached, both PFOA and PFHxA are under investigation. While not included in the Stockholm Convention, short chain PFAS are of growing concern, since manufacturing focus has shifted to shorter chain compounds, and these compounds tend to be present in water, due to their poor sorbtion to soil [16].

Prior studies have indicated potential health effects from PFAS exposure to include low infant birth weights [17], changing cholesterol [18], reduced immune response for vaccinations [19], and thyroid disruption [20]. The Environmental Protection Agency (EPA) included PFOS and PFOA in the third Unregulated Contaminant Monitoring Rule (UCMR3) to determine the scale of contamination across the United States [21]. The data were collected from 2013–2015 and they served as a baseline of likely exposures that were used to inform potential regulatory decisions. In 2016, based on new research, the EPA issued a drinking water health advisory for levels 70 parts per trillion of PFOS and PFOA, individually and combined [22]. The 2016 drinking water health advisory significantly lowered the 2009 provisional health advisory values of 200 parts per trillion of PFOS and 400 parts per trillion of PFOA [22]. By creating a lower, combined health advisory level, water systems previously below the bounds of the 2009 advisory may no longer meet the more stringent guidance. Traditional removal techniques for water contaminants, such as flocculation, sand filtration, or photodegradation without additional chemicals are ineffective against PFAS [23,24]. To combat high levels of long chain PFAS, water systems install activated carbon filters or reverse osmosis systems, both of which are costly [25].

Across the Department of Defense (DOD), PFAS have been included as part of Class B firefighting foam, a type of AFFF that meets military standards [26]. Class B firefighting foam has been in use since the 1960s as both part of suppression systems within a hangar, as well as for training exercises [27]. Incidental runoff from these areas introduced a plethora of PFAS into the surrounding soil. The predominate PFAS of concern in legacy AFFF is PFOS, though PFOA is often present in lesser amounts as it was a contaminant commonly found in the finished product. Once PFOS has reached the soil, it is strongly sorbed leading it to leach slowly into the water table; however, PFOA is not sorbed as strongly as PFOS and shorter chain PFAS are even less well, leading to a faster transit to the groundwater [28]. PFOS plume movement is slow and often takes decades, as demonstrated at Wurtsmith and Tyndall Air Force Bases (AFBs) [29]. AFFF use is common enough to DOD sites that it has been shown there is a 35% increased chance of PFOS contamination within the watershed if a military base is present [30].

At a sufficiently large installation, the U.S. DOD is the water purveyor within those boundaries. The potential water customers include military members and their families who live in government housing, as well as any civilians whose work require them spend their days on site. While it is known that PFOS and PFOA concentrations in groundwater do not show large variance over time [28,31,32], it is not known if small variations occur from month to month. Considering the prevalence of AFFF at DOD sites, if small temporal variations of PFOS and PFOA concentrations in groundwater occur, due to weather patterns, it could place an extra burden on the water filtration system throughout the year. The aim of this study was to determine if PFOS or PFOA concentrations change monthly. If a trend is present, it will also better inform the water purveyors—so they can pay close attention effluent concentration and monitor their treatment beds for signs of breakthrough, due to higher than usual PFAS concentrations. To this end, two Air Force sites with prior legacy AFFF use are examined for changes in the PFOS and PFOA concentrations within the groundwater from month to month.

## 2. Materials and Methods

Data were derived from two sources: Historical data from former Pease AFB and recently collected data from a contaminated military base in Alaska.

PFAS data from former Pease AFB were downloaded from the publicly available Portsmouth Water Works site. Due to widespread contamination, four production wells were monitored monthly for 3.5 years while 13 sentry wells were monitored monthly from between 4 months and 3.5 years [33]. Data were generated by consulting firms employed by the Air Force Civil Engineer Center under the oversight of the EPA. Samples were analyzed by an accredited laboratory that used EPA method 537 for the determination of PFAS in water [34]. The minimum detection limit provided by the lab was no higher than 5 nanograms per liter [35].

Monthly samples from a military installation in Alaska were collected from July 2016 through March 2017. Six wells from around the installation were sampled each month. The wells were selected based on historical sample data that indicated contamination of PFOS and PFOA was present. Water that had passed through granulated active charcoal beds and was ready for distribution was also monitored, denoted as FG. During collection, a new set of gloves was donned, all clothes worn to the site were previously laundered several times, and no cosmetic or lotions were worn. In keeping with the guidance provided in EPA document 600/R-08/092, samples were collected in one-time use polypropylene bottles, immediately put on ice and shipped overnight to a third party lab. Care was taken to avoid contact with Teflon or other known sources of PFAS.

Analysis of samples was carried out through Pace Analytical, a lab certified by the EPA to analyze compounds found in the UCMR3 list. Samples were evaluated using the EPA 537 method using liquid chromatography/tandem mass spectrometry. Each batch of samples were submitted with a field blank to ensure contamination did not occur from improper sampling technique or ambient PFAS levels. The adjusted method detection limit for PFOS was 8 ng/L and for PFOA was 0.4 ng/L. Each batch of samples were reported with quality control data, which included a method blank, a spiked laboratory control sample, and laboratory control sample with duplicate.

In order to determine if there was a relationship between sampling date and PFOS or PFOA concentration, a polynomial (cubic) regression was used. Polynomial regression is an extension of simple linear regression, which includes, in addition to the linear term, squared and cubed terms of the predictor variable [36]. This extension of simple linear regression allows for potential cyclical changes to also be observed. The regression line minimizes the sum of the squared differences between the observations and the predicted value. The null hypothesis is that there is no relationship between date and concentration. To evaluate the hypothesis with the correlated data, the Newey-West variance estimator was used [37]. This estimator was used, because samples were taken from the same wells, which are on the same aquifer, so they cannot be treated as truly independent. The behavior of one well can impact the other, depending on distance and how much one well draws. Therefore, the usual variance estimator, which assumes independence among observations, is inappropriate. The variance was expressed as an *R*^2^ value, which is a number that summarizes how well the independent variable (collection date) accounts for variation in the concentrations. The *R*^2^ value ranges from zero to one, where one describes a perfect relationship.

## 3. Results

Data for PFOS and PFOA concentrations for each site are presented by analyte. A separate cubic regression plot is shown for Pease and for the Alaskan installation. Each month, the concentration of PFOS or PFOA for every well sampled is plotted vertically. Individual wells are denoted by a uniquely colored circle. Each plot contains a red line representing the cubic regression line generated from all the data collected at the site. Wells could not be considered independent, since they drew from a single aquifer. A dashed green line is shown to indicate the current public health advisory stipulated by the U.S. EPA.

### 3.1. PFOA Data

**Figure 1:**
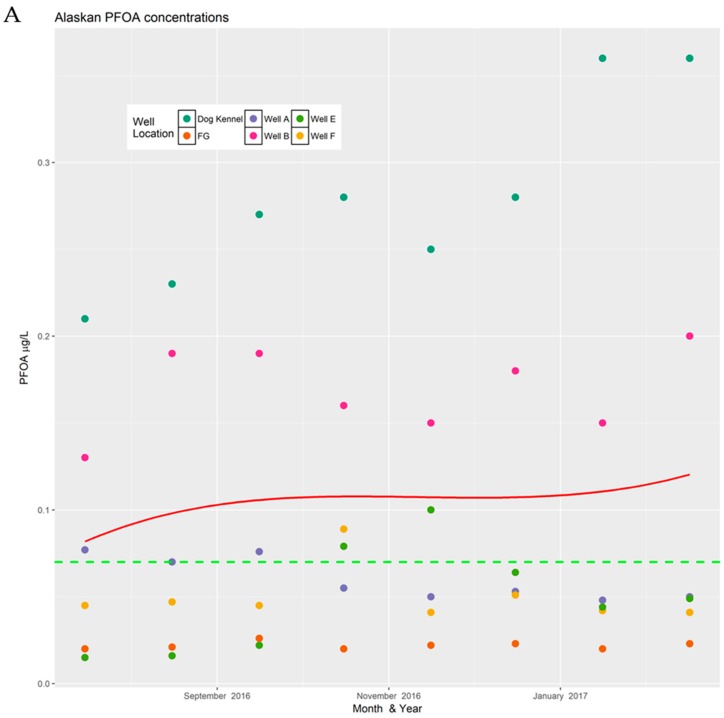

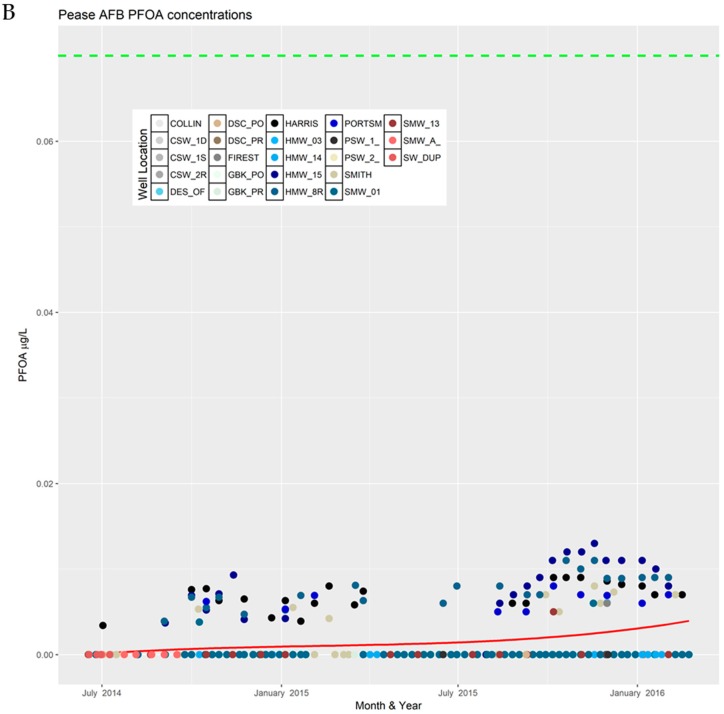
The above chart shows the variance in perfluorooctanoic acid (PFOA) concentrations at two different military installations. (**A**) Alaska military base; (**B**) Pease Air Force Base (AFB). Missing data were excluded from the plots.

PFOA concentrations in each well were plotted by month (Figure 1). Tabular data are available in Supplementary Materials (Tables S1 and S2). Overall, the values at Pease AFB were an order of magnitude lower than those found at the Alaskan site. When comparing variation in all of the wells collectively over time, there was no association between collection date and PFOA at the Alaskan installation (*p* = 0.83). At Pease, the relationship was statistically significant (*p* < 0.001). As can be seen in the plot below, there is considerable spread in the observed concentrations around the fitted line. It is expected that if time played a large role in the concentration changes, the slope of the regression line would be more pronounced and the date would explain more of the variation; however, in the Pease data, the date explained only 10% of the variation in PFOA concentrations (*R*^2^ = 0.10); thus, 90% of the variation could not be accounted for by collection date.

### 3.2. PFOS Data

**Figure 2:**
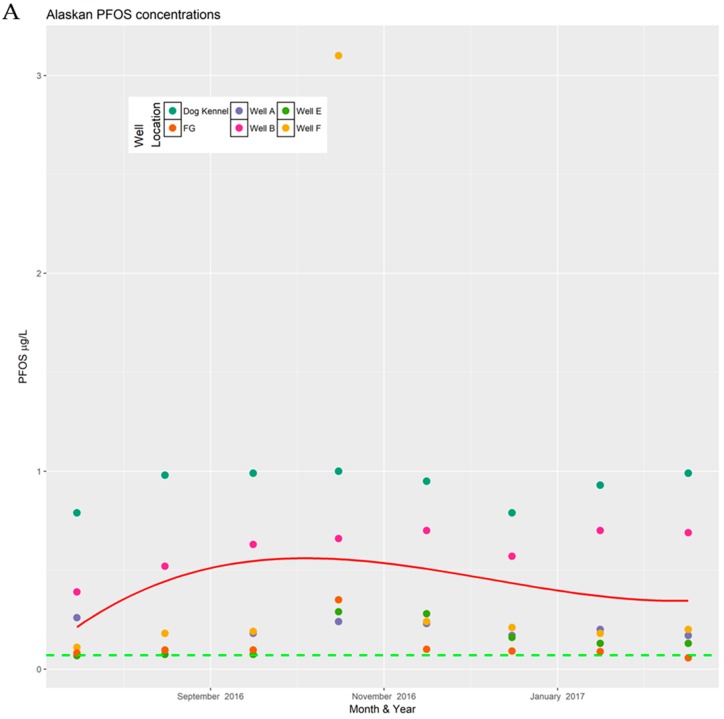

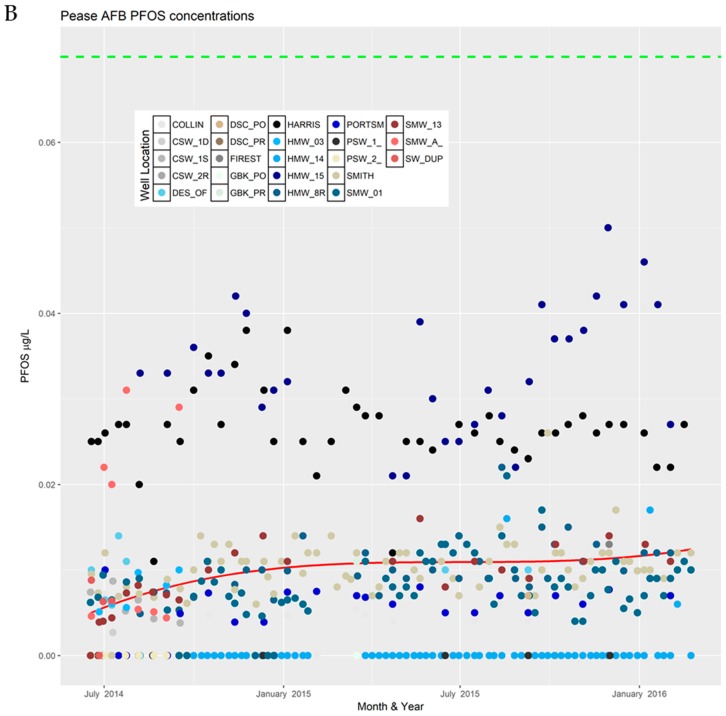
The above chart shows the variance in perfluorooctanesulfonic acid (PFOS) concentrations, over 8 months at, two different military installations. (**A**) Alaska military base; (**B**) Pease AFB. Missing data were excluded from the plots. Because of the numerous data points for the Pease data, only points representing concentrations above 0.02 μg/L were labeled on the plot.

PFOS concentrations in each well were plotted by month (Figure 2). Tabular data are available in Supplementary Materials (Tables S1 and S2). The relationship between collection date and PFOS at the Alaskan installation was non-significant at (*p* = 0.49), while at Pease AFB the relationship was statistically significant (*p* < 0.001), but explained little of the variation in PFOS concentrations (*R*^2^ = 0.04), leaving 96% of the variation unexplained by the collection date. These values are higher than the reported PFOA concentrations, suggesting the primary source of contamination at the Alaskan site is from AFFF. Previous studies have found a similar pattern in concentrations when studying AFFF [38,39].

Concentrations at five of the wells sampled at the Alaskan installation were above the EPA health advisory level for at least one measurement. Interestingly, PFOS concentrations were consistently highest at the dog kennel well, followed by well B. This is likely due to the proximity of the wells to point sources, caused by prior firefighting exercise.

For wells A, E, and F, the concentrations were above the health advisory level for one to two out of eight measurements. Therefore, the use of a single annual measurement should consider the possibility for random variation in the reading that could affect future EPA compliance. The total span in the measured concentration across months was 29, 222, and 2990 ng/L for wells A, E, and F, respectively.

**Figure 3:**
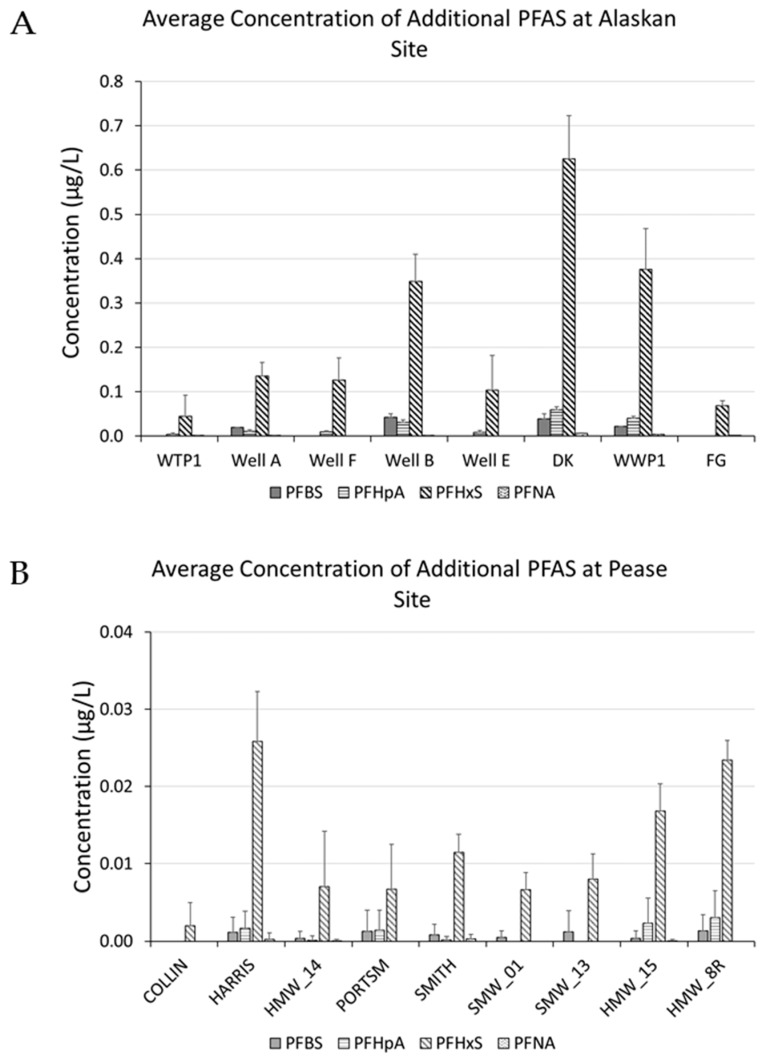
The above chart shows the average and standard deviation in additionally sampled polyfluoroalkyl substances (PFAS) concentrations during the same time frame. (**A**) Alaska military base; (**B**) Pease AFB. Missing data were excluded from the plots. Because of the numerous data points for the Pease data, only wells that consistently had positive identification of the PFAS are shown. PFBS, perfluorobutanesulfonic acid; PFHpA, perfluoroheptanoic acid; PFHxS, perfluorohexane sulfonic acid; PFNA, perfluorononanoic acid.

Data on perfluorobutanesulfonic acid (PFBS), perfluorohexane sulfonic acid (PFHxS), perfluoroheptanoic acid (PFHpA), and perfluorononanoic acid (PFNA) were collected (Figure 3). When reviewed, if the analytes were present, they showed similar trends to those observed with PFOS and PFOA.

## 4. Discussion

Based on the statistical model detailed above, there was not a statistically significant correlation between PFOS and PFOA concentration and collection date at the Alaskan installation. At Pease AFB there were statistically, but not practically significant correlations. As can be seen by the wide variation in observed concentrations above and below the collection date concentration regression line, collection date is of little value in predicting contaminant concentrations, as one would expect given *R*^2^ values of 0.10 and 0.04 for PFOA and PFOS, respectively. Therefore, the Pease results have no practical importance and monthly sampling would not be useful for wastewater or drinking water managers in the monitoring of PFOS or PFOA. This is similar to the results of a previous study that found seasonal trends were not observed in PFOS and PFOA concentrations from wastewater treatment facility effluents [40]. This result is also compatible with the findings published in literature that showed long chain PFAS concentrations vary insignificantly over years [41]. While not part of current EPA health advisories, the concentration of PFHxS observed was comparable to the PFOS concentration at both sites. PFHxS is currently under consideration by the Stockholm Convention for classifying it as a persistent organic pollutant.

The United States Government Accountability Office published a report in October 2017 outlining the state of the DOD response to PFAS contamination [42]. As of May 2016, the military departments of the DOD have all restricted use of legacy AFFF to emergencies to avoid unnecessary releases into the environment. Testing of alternative AFFF without PFOS as a component is in progress by the Navy. DOD funding is currently being used to explore AFFF that does not use PFAS at all, although testing is anticipated to take until 2020. This final item, the investigation of AFFF that does not require PFAS to operate, is paramount, since there is concern that the proliferation of replacement PFAS only differs minutely from PFOS and PFOA [43]. Within the European Union, AFFF that contains PFOS was banned in 2011. Other PFASs have been identified as substances of concern, under the European chemicals regulation, REACH. A number of PFOS and PFOA AFFFs are available, though adoption of any of these alternatives by the U.S. DOD must follow a rigorous review.

There were significant limitations to this study. Both Pease and the Alaskan military installation draw their water from a single aquifer, so wells within the field cannot truly be treated as independent from one another, complicating analysis of the concentration changes observed. The longest monitored site in Pease was 2.5 years. Ideally, the study would include five years of data collected at two-week intervals to produce more conclusive results. Both time and financial constraints did not allow for this reality.

## 5. Conclusions

PFAS are persistent, pervasive contaminants that have been found on all continents and in the serum of many animals that have been studied [44,45,46]. They resist degradation while in the environment and require more sophisticated purification techniques to remove from water, once they have been introduced [25,47,48]. There is a growing body of evidence to suggest low-level exposure to PFAS has long-term effects on humans [49,50]. The inclusion of PFOS and PFOA in the UCMR3 and the reduction in suggested concentration for the EPA’s drinking water health advisory have given these compounds public visibility in the United States [51,52,53].

The results of this study agree with the consensus for short-term laboratory tests that PFAS concentrations do not vary significantly on the scale of weeks or months, although the results presented here are drawn from true environmental samples [54]. Conversion from traditional water treatment techniques to more sophisticated techniques is an expensive undertaking. In the United States, the Defense Environmental Restoration Program requires the DOD to clean up current and former installations, including remediating contaminated land after it has been transferred to another party if contamination is discovered after the transfer has taken place [42].

The Government Accountability Office report also outlines the number of current and former military installations suspected to be contaminated with legacy PFAS: 203 installations in the Air Force, 127 installations in the Navy, and 61 installations in the Army [52]. As new AFFF are introduced, the risk of exposure to different PFAS than PFOS or PFOA increases. Comprehensive monitoring of PFAS is needed, since human health effects for shorter chain PFAS is still of concern. No one-size-fits-all remediation strategy exists. Several competing in situ mediation techniques exist in varying stages of development [55,56]. Some water treatment techniques are mature enough to treat contaminated sites prior to distribution to consumers, but these treatments do not address the underlying contamination. The longevity of these contaminants in the environment requires strategic treatment for individual sites. The Air Force has identified more potentially contaminated sites than both the Navy and Army combined, so proactive investigation is critical in the coming years.

## Supplementary Materials

The following are available online at http://www.mdpi.com/2305-6304/6/3/56/s1, Table S1: PFOA concentrations in ng/L at former Pease AFB; Table S2: PFOS concentrations in ng/L at former Pease AFB.
